# Gene Regulatory Network Reconstruction Using Bayesian Networks, the Dantzig Selector, the Lasso and Their Meta-Analysis

**DOI:** 10.1371/journal.pone.0029165

**Published:** 2011-12-27

**Authors:** Matthieu Vignes, Jimmy Vandel, David Allouche, Nidal Ramadan-Alban, Christine Cierco-Ayrolles, Thomas Schiex, Brigitte Mangin, Simon de Givry

**Affiliations:** SaAB Team/BIA Unit, INRA Toulouse, Castanet-Tolosan, France; University of Sheffield, United Kingdom

## Abstract

Modern technologies and especially next generation sequencing facilities are giving a cheaper access to genotype and genomic data measured on the same sample at once. This creates an ideal situation for multifactorial experiments designed to infer gene regulatory networks. The fifth “Dialogue for Reverse Engineering Assessments and Methods” (DREAM5) challenges are aimed at assessing methods and associated algorithms devoted to the inference of biological networks. Challenge 3 on “Systems Genetics” proposed to infer causal gene regulatory networks from different genetical genomics data sets. We investigated a wide panel of methods ranging from Bayesian networks to penalised linear regressions to analyse such data, and proposed a simple yet very powerful meta-analysis, which combines these inference methods. We present results of the Challenge as well as more in-depth analysis of predicted networks in terms of structure and reliability. The developed meta-analysis was ranked first among the 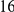 teams participating in Challenge 3A. It paves the way for future extensions of our inference method and more accurate gene network estimates in the context of genetical genomics.

## Introduction

### Inferring gene regulatory networks

Gene regulatory networks (GRN) are simplified representations of mechanisms that make up the functioning of an organism under given conditions. A node in such a network stands for a gene *i.e.* a DNA fragment that encodes a functional agent of the cell such as a protein. Proteins are among the most well-studied acting protagonists in living organisms. In large part, their synthesis is effectively regulated by other interacting proteins. In a GRN, edges depict causal relationships between sources and targets for gene activities. Hence a convenient representation for GRNs are directed graphs. The objective of the third DREAM5 challenge was to infer causal relationships in artificial complex networks.

More generally, a biological network is defined by constituents at different levels, such as DNA sequences, RNAs (messengers, but also small RNAs), proteins, metabolites. Discounting epigenetic effects, genes barely interact directly. They are rather activated or repressed through the action of specific components acting at other scales [1]. The work presented in this paper is focused on gene regulatory interactions. This representation maps the action of all cellular components onto gene space. Potential applications still benefit from this simplified interpretation of the complex system. For example, the search of candidate genes that target changes in a phenotype of interest [2], the study of evolutionary aspects of biological networks [3], [4] so as to link their structure to functional properties [5], [6] all use the representation of gene regulatory networks.

Initially, specific attention has been devoted to understanding the dynamics [7] and principles governing gene regulation, using either the first rules in logic to capture the absence or presence of cycles in a Boolean formalisation of a GRN [8]. Later, [3], [9] also studied the successive refinements on gene network topology and their functional consequences. In the past ten years, motivated by the abundance of micro-arrays, a huge effort has been devoted to GRN inference. The methods that were proposed and developed include analyses based on correlations in the data [10], systems of ordinary or partial differential equations that give a plausible physico-chemical modelling [11], systems of linear equations [12] and Bayesian networks (BN, [13]) to cite only a few. Additional improvements were proposed depending on the exact nature of the data at hand (*e.g.* time series, [14]–[16]) and biological *a priori* knowledge [17].

### Genetical genomics paradigm for gene network inference

In order to decipher causal links, the above-cited methods rely on expensive and still technically challenging time series data or on many experiments perturbing the systems from a steady state (*e.g.* by studying the effect of knocking out a gene). “An ideal experimental design for causal inference is randomised, multifactorial perturbation” recalls the website of the third challenge of the Dialogue for Reverse Engineering Assessments and Methods (DREAM5, [18]) giving a makeover to Fisher's work on experimental design [19] in molecular biology data analysis. Genetic polymorphisms in a segregating population are ideal settings for multifactorial perturbations of a living system: each allele is a potential source of perturbation for network behaviour. Recombination and segregation events that occur during genetic crosses, randomise the distribution of these alleles among the lines derived from two genetically known and diverse parents. Systems genetics, or more precisely “genetical genomics” [20], [21], is the study of how such randomised genetic perturbations can directly or indirectly affect numerous complex traits. These traits can be either qualitative phenotypes of interest or quantitative measurements reflecting the activity of cells like transcriptomics data. The variety of patterns in trait responses on genotyped individuals in the segregating population are used to draw causal inference. The added value of having both genetic and perturbed phenotypic (expression) data has already been demonstrated, in particular to infer causality [22]. Existing works that elucidate GRN structure based on genetical genomics data have been using Bayesian networks (BN) using genetic data as prior information [23] or multivariate regression in a structural equation modelling (SEM) framework with multiple testing and greedy search steps [24]. Their efficiency and accuracy in dealing with high dimensional data set is still very limited.

In this paper, we consider complementary approaches that could potentially improve over state-of-the-art methodologies to perform GRN inference from systems genetics data sets, namely (i) penalised regressions: Lasso [25]) and the Dantzig Selector [26] which seek linear interpretable dependencies with a controlled level of parsimony and (ii) BN with an appropriate scoring function and an integrated treatment of genetic and genomic data. These approaches are used as inputs to feed a consensus statistical meta-analysis approach that combines the best of other learning algorithm results, and which emerged as the best performer for the DREAM5 Challenge 3A on GRN inference in systems genetics. Since there is still no large experimental data set for which a reliably known large size gene network exists, the challenge offered simulated data based on differential equation simulation, defining Gold Standard data sets.

The first section of the paper is devoted to the results we obtained. A discussion on the relative merits and limitations of the proposed methods follows. The “Material and Methods” section precisely describes the data and the different methods used, including specific adaptation to the data sets and post-processing used to produce network estimates.

## Results

In this section, we present our results and the prediction performance achieved according to the DREAM5 challenge criteria and we then give a more in-depth analysis in order to gain more insight on learnt GRNs. The data sets provided by the challenge organisers, which are described in more detail in the “Material and Methods” section, contained simulated genotypes in recombinant inbred line (RIL) populations of variable size (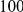, 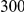 or 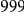 individuals) and their associated expression levels, which were governed by inductive or repressive effects of genes on each other according to the topology of plausible networks to recover. For each RIL population size, five networks with an increasing number of edges were simulated, so a total of 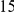 data sets were provided.

Since the meta-analysis that we used was the best performer of the challenge, we focus on the results obtained using this consensus method. To illustrate the complementarity of the different methods (BN, Lasso and Dantzig-based regressions) that supplied input edges to the meta-analysis, we also present several aspects of their predictions. According to DREAM5 specifications, a predicted network topology is defined by a list of directed edges ranked according to a non-increasing order of confidence score.

### General results

Edge lists were compared both to (i) Gold Standard files, namely the correct list of edges used in simulated models and to (ii) the pool of all edges that were submitted by other participating teams.

**Figure 1:**
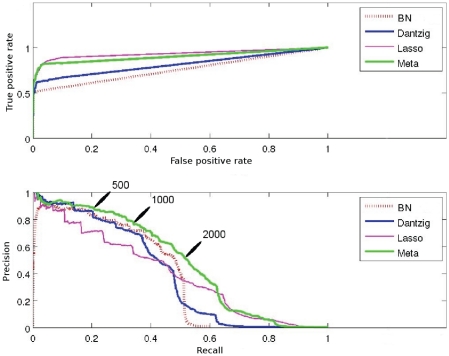
Accuracy results for the GRN inference methods: ROC curves (upper panel) and PR curves (lower panel) for Network1-A999. Meta-analysis: green, BN: dashed red, Lasso: purple and Dantzig: blue. Points for inferred networks of 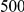, 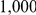 and 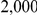 edges are indicated.

Receiver Operating Curves (ROC *i.e.* True positive vs. False positive rates – FPR) and precision vs. recall (PR) curves were produced for each network. The false positive rate assesses the trend of the method to produce incorrect edges. The recall is equal to the true positive rate and measures the power of a method to recover the complete set of true edges. The precision (the rate of correctly made predictions) is an indicator of the reliability of the predictions. Curves obtained by the meta-analysis, BN, Lasso and Dantzig approaches on the sparsest network, with 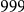 individuals (Network1-A999), are shown in Figure 1.

In ROC curves, the point FPR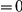, recall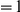 corresponds to an ideal situation where all and only correct edges would be predicted. This ideal point was not reached by the meta-analysis, however an interesting trade-off value of (FPR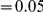, recall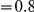) was reached. The excellent FPR value was not surprising given that the simulated networks were rather sparse. The steep slope at the beginning of the ROC curve is a good indicator that the edge ranking produced by the methods was reliable.

The PR curve plots the ability of the methods to produce both reliable and comprehensive predictions. For example, with the 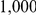 first edges obtained by the meta-analysis, a precision value of 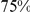 means that three out of four inferred edges were correct, whilst the recall value of 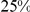 means that one out of four original edges was recovered.

It should be noted that since the Lasso and Dantzig approaches produced up to 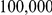 edges, so did the meta-analysis. The BN method produced sparser edge lists: between 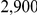 and 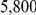 edges per network. This makes the reliability of the score assigned to edges a key point: no one is really interested in predicting a true edge that is ranked 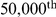. In the next section, detailed features about our results are presented, and the emphasis is put on how these features can serve GRN inference when a Gold Standard network is unknown.

Table 1 presents the area under the ROC and PR curves (AUC) of the 15 inferred networks for the meta-analysis approach. Results clearly showed that reducing the size of the sample made the problem much harder. At the same time, it also appeared that increasing the edge density of the simulated network (from Network ‘1’ with 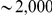 edges to Network ‘5’ with 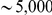 edges) also made the challenge of GRN inference slightly more difficult, since prediction performances decreased.

**Table 1:** AUC of the DREAM5 Challenge 3A for the meta-analysis of the SAaB team (source: [18]).

DREAM		Area Under the Curve (AUC)				
challenge		Network ‘1’	Network ‘2’	Network ‘3’	Network ‘4’	Network ‘5’
A999	PR	0.358^a^/0.482^b^	0.258/0.364	0.195/0.292	0.183/0.260	0.178/0.244
	ROC	0.933/0.902	0.885/0.845	0.844/0.808	0.821/0.784	0.813/0.768
A300	PR	0.211/0.248	0.144/0.175	0.141/0.159	0.132/0.141	0.113/0.131
	ROC	0.855/0.845	0.793/0.779	0.786/0.774	0.759/0.739	0.737/0.719
A100	PR	0.085/0.074	0.060/0.054	0.053/0.045	0.054/0.046	0.054/0.044
	ROC	0.754/0.750	0.718/0.713	0.696/0.694	0.676/0.671	0.670/0.666

^a^AUC official values issued by the DREAM organisers.

^b^AUC after minor corrections in our implementations.

Since the publication of the official results of the DREAM5 challenges, we have slightly improved the post-processing of our approaches. For example, the handling of edge direction is now identically dealt with by the two penalised regression approaches. Consequently, the meta-analysis AUC also changed. PR trade-offs were noticeably improved, whilst ROC slightly decreased.

The prediction of every method against the pool of all the predictions submitted by the teams that entered the challenge was also assessed. It was used to produce empirical 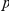-value derived scores [27] that reflect how good each method performed in comparison to others and was eventually used to rank teams. Our meta-analysis method achieved first place in the three sub-challenges 3A (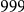, 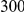, 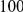 individuals) with respective overall scores of 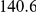, 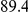 and 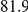. In sub-challenge 3A999, our scores were the best for both ROC and PR scores. In sub-challenge 3A300, two different teams provided better results: one for the ROC curve and one for the PR score, although none of them achieved a better overall score than our meta-analysis.

### Detailed results

In this section, we present a detailed analysis of the results obtained on the most favourable case, which is Network 1 with 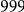 individuals (Network1-A999). This choice is naturally arguable, since a common situation in a systems genetics context is to infer relationships between genes when sample size is limited. It however gives an upper bound on the performances achieved on all networks and defines an ideal situation where the most reliable observations and conclusions can be drawn.

#### Correct edges come first

Since predicted edge lists can be as long as 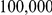 edges and since we are interested in obtaining reliable and interpretable predictions only, we focus on the first 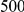, 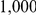, 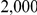 and sometimes 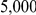 edges. The ‘Results’ section established that such short-list of predicted interactions simultaneously gave reasonably good coverages and acceptable precision levels (see corresponding precision and recall values in Figure 1). Moreover, they represent sets of edges whose sizes are reasonably manageable in the context of a 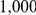 gene regulatory network that must be deciphered without any prior knowledge.

We tried to infer the directed network topology from the 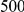 first edges of the meta-analysis. 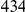 of them (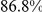) were correct, but 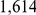 edges among the 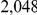 edges of the true network were missing. So the recall was only 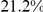. When we used the 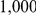 or 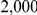 first edges, the recall increased to respectively 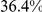 and 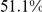 but the price to pay was a drop in precision to respectively 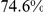 and 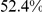. So inferring half of the true network led to an inference noise of nearly 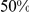.

For denser networks, the precision remained the same, but the recall decreased since the total number of edges to predict was greater. As an example, in Network 5 (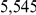 regulatory relationships), the 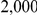 first edges produced by the meta-analysis had a precision rate of 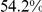 but the total network coverage was only 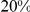. In this case, raising the total number of edges to keep for inference purpose was not a good option since using the 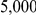 first edges indeed slightly increased the recall to 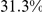, but the precision then went down to 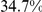 (data not shown).

#### In/Out-degree distributions

**Figure 2:**
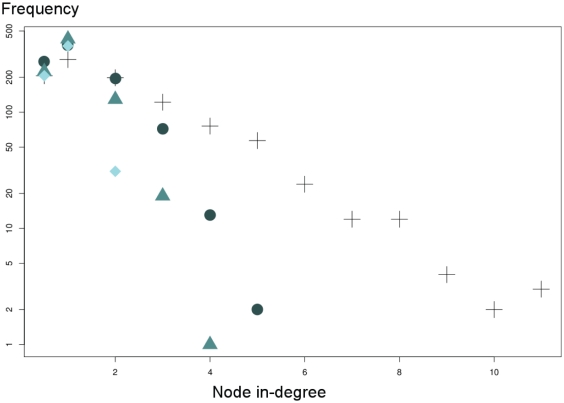
In-degree distribution of Network1-A999. The distribution is plotted on the log scale on the 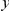-axis since the in-degree distribution was assumed to be exponential in the true network (black crosses). Coloured symbols stand for the first 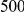 (light green diamond shape), 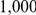 (middle green triangles) and 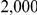 (dark green circles) edges inferred by the meta-analysis.

**Figure 3:**
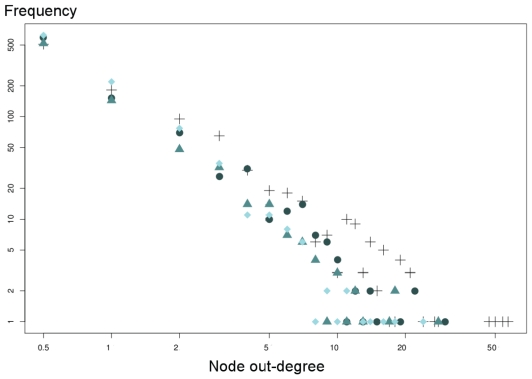
Out-degree distribution of Network1-A999. The distribution is plotted on a log-log scale since it was expected to be a power-law distribution in the true network (black crosses). Coloured symbols stand for the first 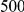 (light green diamond shape), 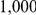 (middle green triangles) and 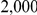 (dark green circles) edges inferred by the meta-analysis. Points having ‘
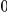
’ out-degree were transformed to 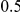.

If initially the only knowledge that was available on the simulated networks was that they had a modular structure, the organisers later revealed how they were generated, including simulated in/out degree distributions. It is therefore informative to compare Gold Standard networks to predicted networks in terms of node degree distributions. Figures 2 and 3 compare plots of respectively in- and out-degree distributions for the true Network1-A999 and for networks inferred from the first 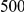, 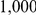 and 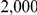 edges predicted by the meta-analysis. The first result was that the larger the set of edges, the more accurate the predicted network topology: inferred degree distributions got closer to the correct ones when the edge list was increased. This would obviously not be true if we had considered much longer edge lists (which have poorer precision levels): a list of tens of thousands of edges would give too high a network connectivity, and degree distributions would be skewed. With the number of edges that we considered, distributions were skewed towards 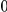 and some nodes were isolated even when 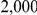 edges were kept (see the paragraph on largest connected components, below).

**Figure 4:**
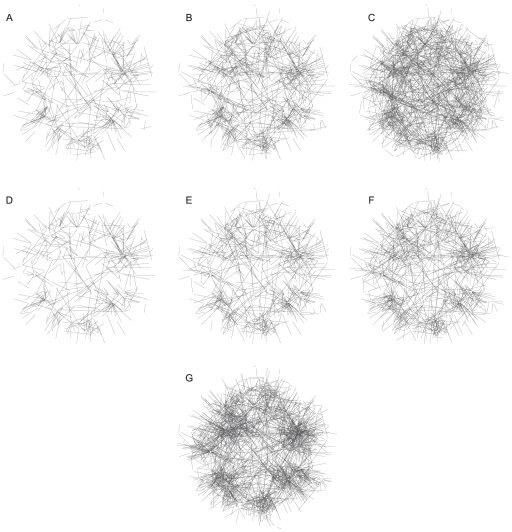
Network1-A999 visualisation. (A) to (C) are networks inferred by the meta-analysis using the first 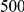 (A), 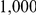 (B) and 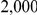 (C) edges. (D) to (F) represent the same predicted networks showing only correctly inferred edges. (G) is the true network. For clarity, vertices have been removed.

The in- and out-degree distributions of the true network and its modularity are global essential features of its topology. This modular structure appeared with as few as the first 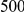 edges and was clearly visible with the first 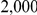 edges, as it is illustrated in Figure 4. However, the method had difficulties in locally capturing relationships for a node that had many incoming links: it was quite difficult to unmask regulatory hubs. For example, the true network had a dozen genes with more than 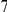 incoming edges and our predictions among the first 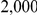 edges revealed only one node with 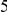 incoming links. Moreover, the true network had one 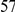-outgoing relationship hub and using the 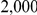 first meta-analysis edges, we predicted only 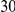 such links for this hub. A consequence of this difficulty in predicting highly connected nodes was that our predictions overestimated the number of nodes with few regulatory connections.

Despite this, the meta-analysis performed relatively well at inferring networks with relatively accurate in- and especially out-degree distributions. In real biological data sets applications, if one had some prior knowledge about the true degree distributions (*e.g.* from another well-studied organism) plotting inferred node degree distributions would probably be a good tool for assessing network overall quality.

#### Largest connected component

**Figure 5:**
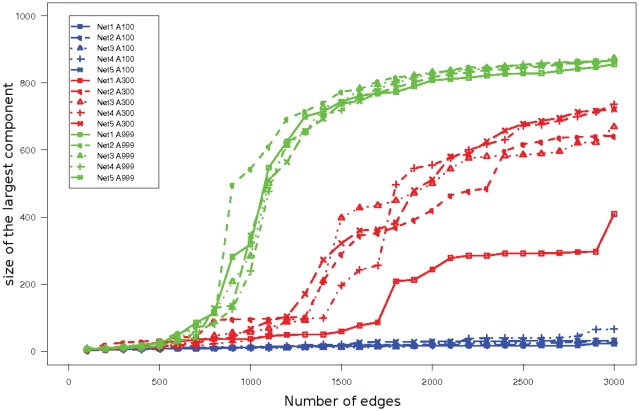
Size of the largest connected component inferred by the meta-analysis for the 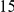 DREAM5 Challenge 3A networks vs. number of edges. Colours encode sample sizes: blue for 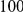 individuals, red for 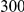 and green for 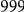. Line style and symbols on curves represent networks: solid line squares for Networks ‘1’, short dashed line with circles for Networks ‘2’, dotted line with triangles for Networks ‘3’, alternate dashed and dotted line with plus for Networks ‘4’ and long dashed line with crosses for Networks ‘5’.

In the considered Gold Standard 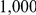 gene networks, all nodes were connected. Moreover, in real biological data sets, it is often acknowledged that a GRN has a giant connected component [28] *e.g.* for robustness reasons. So being able to predict such a structure is a positive point for an inference method, even if all interactions are not simultaneously active [29]. The previous analysis on in-/out-degree also suggested to look at the size of the largest connected component when the number of considered edges increased. Figure 5 shows how this size evolves with the number of considered edges for the 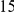 different networks of the challenge. Clearly, three trends appear in our results, depending on sample size. In the 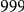 individuals case, the largest connected component captured almost all nodes when more than 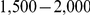 edges were considered, whatever the true graph connectivity. In the 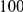 individuals case, no large connected component appeared, even when considering 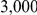 edges (with very low precision levels near 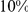). For 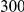 individuals, it was a middle-of-the-road case. A large connected component appeared reasonably quickly with additional edges, but the behaviour changed with the true network connectivity.

Up to this point, we only presented global measures on the networks. In the next paragraph, we present results that show that prediction accuracy may also be influenced by local factors such as the type of mutation that occurred, either in the promoter region or in the coding sequence of the gene.

#### Edge inference accuracy depends on mutations that impact gene activity

We analysed the quality of inferred gene regulations depending on the type of mutation that occurred for the source (regulator) gene and the target (regulated) gene. We inferred the type of mutation of a gene: we labelled the gene ‘*cis*’ if the mutation is in its promoter region (hence the mutation shows a *cis*-regulatory effect), and ‘*trans*’ if it lies in its coding region. A *trans*-mutation modifies the sequence of the gene which, as a regulator, affects the expression of target genes in the GRN. This leads to a *trans*-regulatory effect. Some authors (*e.g.* [24]) call such regulation a ‘*cis-trans*’ effect, but we used ‘*trans*’ for simplicity.

For each gene, we tested the *cis*-regulatory effect of its marker using an analysis of variance, as described in the “Materials and Methods” section (Bayesian networks subsection). Genes not detected as *cis*-regulated were labelled ‘*trans*’. This gave a predicted number of *trans*-acting genes consistent with the announced frequency of 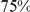 when the sample size was large enough to precisely infer this rate. When sample size was smaller, we underestimated *cis*-acting regulation frequency.

**Figure 6:**
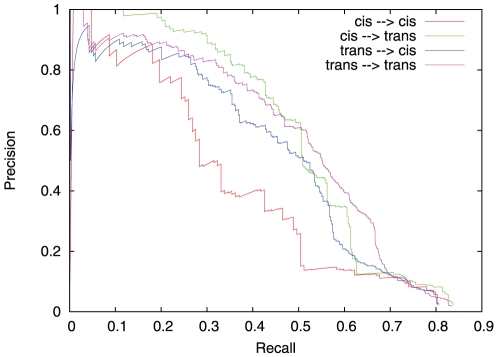
Analysis of precision/recall of the meta-analysis approach for DREAM5 Challenge 3A with 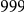 individuals/Network1 data. Predicted gene regulations are classified into four groups depending on the label of the regulator and the target gene. A gene is labelled ‘*cis*’ if its marker has a *cis*-regulated effect on its expression level. Otherwise, gene is labelled ‘*trans*’. An edge between two ‘*cis*’ labelled genes is classified ‘*cis* 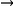 *cis*’, between two ‘*trans*’ labelled genes: ‘*trans* 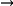 *trans*’ and so on.

Figure 6 shows that ‘*cis*’ 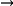 ‘*trans*’ links were predicted more reliably than other types of relationships. This may be explained by the fact that the regulator of the target gene had a large variation due to the strong effect of its *cis* mutation, and that its regulatory effect was not obfuscated by a *cis*-regulation on the target gene. The ‘*cis*’ 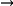 ‘*cis*’ framework was the worst from the prediction accuracy point of view. It may correspond to strong correlations due to genetic linkage but not to direct causal regulations.

#### Complementarity of the inference methods combined in the meta-analysis

The meta-analysis took as input the inferred networks of three different methods: the Bayesian networks (BN), the Lasso regression, and the Dantzig selector-based regression. These methods ranked the edges differently and this was what allowed the meta-analysis to perform well.

**Figure 7:**
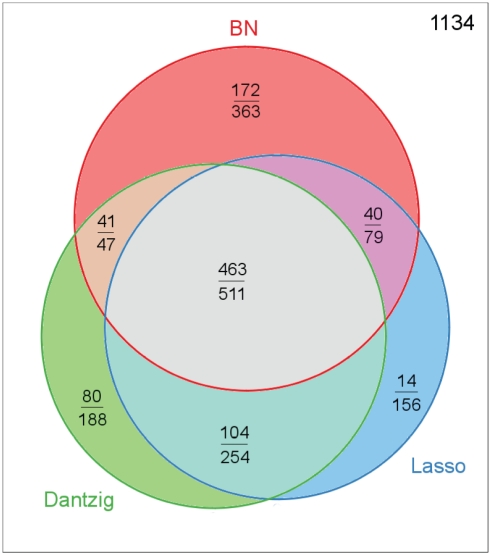
Venn diagram between the three sets made up of the first 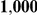 edges inferred from one of the three approaches: BN (red circle), Lasso (blue circle) and Dantzig (green circle). Within each region of the diagram, the number of correctly inferred edges (over the bar) and the total number of edges (under the bar) are given. 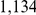 (top right) is the number of missing edges for the union of the three approaches.

Figure 7 displays a Venn diagram that presents specificity and overlaps between the sets of the first 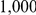 edges predicted by the BN, Lasso, and Dantzig approaches, respectively. Similar figures were obtained with the first 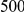 or 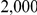 edges instead (data not shown). It appeared that the edges simultaneously predicted by all three approaches were very reliable: 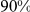 of them were correct. So were edges shared by the BN and Dantzig approaches. Edges predicted by just one method were less precise (less than 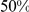 precision), and pure Lasso predictions were even poorer.

The most interesting observation was that there was a clear complementarity between the three considered approaches. They shared a core prediction set, but each of them provided a specific contribution to the meta-analysis output.

#### Computing times

Table 2 presents computing times for the different approaches. These computation times were averaged over the five different networks in each of the three different sub-challenges. Computation times were very similar for the different networks, although the number of edges in the network to infer varied from less than 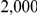 to more than 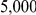. The CPU-times for the BN and Lasso methods had a nearly linear dependency upon sample size and should scale-up easily to larger data-sets. This seemed less obvious for the Dantzig selector approach but this was mostly because this recent method has been directly and bluntly implemented using a general linear programming solver. The use of a dedicated algorithm such as DASSO [30] would likely lead to an approach that scales as smoothly as the Lasso approach.

**Table 2:** Computation times for the different approaches (per network).

Method	DREAM5 sub-challenge		
	A100	A300	A999
BN	20′	70′	180′
Lasso	5′	12′	30′
Dantzig	300′	1300′	6600′
Meta	less than a couple of seconds		

CPU times are given for a 2,96 Ghz Intel(TM) processor with 4 GB memory installed.

The meta-analysis is almost instantaneous as it only needs to parse edges lists for BN, Dantzig and Lasso to produce its own network and edge scores. However, it can not be run independently of other methods.

## Discussion

We have proposed a GRN reconstruction method that relies on a meta-analysis of the output of three different reconstruction methods (namely BN, Lasso and Dantzig). As best performers of the DREAM5 Challenge 3A, we have shown that the presented methodology can adequately deal with large size gene network inference in a systems genetics (or genetical genomics) framework, *i.e.* when both marker data, that reflects mutations occurring in a segregating population, and gene expression data are available.

As expected, network reconstruction clearly improves when sample size increases. This is a decisive argument for planning genetical genomics experiments with enough individuals in the segregating population. Our results suggest that a sample of size 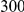 is at least needed to infer a first list of 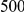 reliable edges (at a precision level of nearly 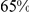) for a 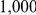 gene network using the meta-analysis approach.

These good results could only be achieved thanks to the integration of three different complementary statistical inference techniques. This is certainly a key explanation for the results obtained. First of all, these predictions were produced by two different classes of methods, each capable of exploiting specific different features.

• structure learning of a Bayesian network: in this probabilistic framework, a directed acyclic graph is used to represent probabilistic relationships between discrete variables. The directed acyclic graph structure restricts the class of predicted GRN to structures that do not contain feedback loops. However, the use of directed graphs allows for predicting causal relationships between variables, as expected in the DREAM5 challenge.

Discrete Bayesian networks are also inherently limited by the usual encoding of probabilistic relationships between causes (parents) and effects using a conditional probability table for each node in the network. Such a table includes a number of parameters that grows exponentially with the number of causes. Since sample size is limited, only a limited number of parameters can be reliably estimated, and the approach is therefore inherently limited to graphs where every variable is explained only through a limited number of parents. For the Bayesian Information Criterion (BIC) a maximum number of 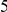 or 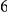 parents, depending on the choice of 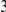 or 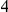 classes for expression level variables, could be predicted with a sample size of 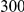 [31]. The BDeu score that we used for the structure learning is known to allow more parents [32], however we never attained the hard constraint of 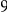 parents that we imposed in our algorithm. This restriction was necessary for computational efficiency, as learning Bayesian network structure is NP-hard [33].

The positive part of this flexible encoding of probabilities distributions is that it enables the capture of non-linear relationships between variables, an expected behaviour of true biological samples.

• penalised linear regressions (Lasso and Dantzig): as a mirror to Bayesian networks, these approaches infer undirected graphs, with no causal relationships, but the predicted structures may contain cycles. They are restricted to linear relationships between variables, but this restriction keeps the number of parameters small. The number of neighbours of a variable is not *a priori* limited, and predicting hubs is possible. Ultimately, these models are efficient in the sense that the associated inference algorithms are polynomial time algorithms [30], [34].

Unexpectedly, despite the relationships between the two penalised linear regression methods [35], which should provide close estimates in a sparse setting, the Venn diagram in Figure 7 clearly shows that each method predicts different sets of edges showing complementarity even in their own class.

The idea of combining results from different methods has already been tested by the DREAM organisers themselves in a previous different DREAM challenge, in what they called “the community intelligence” [36]. With the best performers among the competitive teams, the DREAM organisers computed a a very simple and robust combined score based on rank sum. Their predictions outperformed individual teams when results of best performers were complementary and not optimal. We based our meta-analysis on a more sophisticated score that was accurate because our source methods had weighted edges with a probability-like score. Clearly, combining linear (Lasso/Dantzig) and non-linear (BN) methods allowed the meta-analysis we proposed to better detect causal relationships.

BN and penalised regressions also produced a very different total number of predicted edges. The number of predicted edge has a tremendous impact on DREAM challenge scores. BN predictions hardly reached a few thousand edges whilst Lasso and Dantzig approaches produced more than 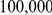 edges each. To illustrate this, the area under the curve for true positive rate versus false positive rate in Figure 1 (top) was clearly smaller for BN predictions. Edge list of smaller length can be an explanation of poorer scores. The meta-analysis used the entire list of scores produced by BN, Lasso and Dantzig approaches. The edge ranking score (described in the “Material and Methods” section) we used gave better results than any of the individual approaches, except for the very first predictions (recall below 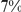 on Figure 1 bottom) ; in this latter situation, the Dantzig approach obtained slightly better precision (less than 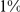 improvement).

General features of the true network are ususally correctly recovered. For example, predicted networks have good in- and out-degree distributions and the expected construction of a big connected was quickly observed with only 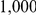 or 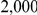 predicted edges. In addition to individually ranking correct edges first, the meta-analysis is also able to retrieve global structural attributes of the network.

One obviously has to be careful about conclusions drawn from simulated data, as provided in the DREAM5 challenge. While experimental data on GRN slowly accumulates and expression measures become increasingly easy and inexpensive using RNA-Seq, to the best of our knowledge, neither sufficiently large experimental data sets that systematically combine gene expression and polymorphism measures, nor experimentally confirmed large GRN are available yet. In the area of genetical genomics data-sets, [37] exploited 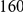 RILs from a cross between two *Arabidopsis thaliana* accessions with 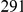 available markers and 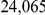 gene transcript levels. Similarly, [38] gathered 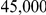 gene expression levels, 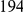 micro-satellite markers for 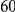 F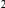 mice and [39] recently analysed 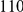 RILs derived from a cross between two rice accessions with 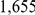 markers and 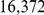 expression traits. The three former examples were far from inferring a genome-scale GRN. These examples stress the gap between present research results obtained on real data sets that give only local regulatory relationships and simulation settings that indicate a potential for genome-scale GRN reconstruction on larger data sets. From our experience in analysing such data sets, several features can be quite different in real data sets and in simulated data sets, such as those proposed in the Challenge 3 of DREAM5.

One such feature is the unrealistic one marker per gene assumption: in practice, the total number of markers is unrelated to the number of genes and may be either quite low, or very high (see [40] for figures on plants and references therein with over a million SNPs for humans). A solution to the former case would be to infer pseudo-markers but still less comprehensive information would result from it. On the other hand, Next Generation Sequencing data sets are promising in that they would propose several markers per gene. Our modelling need be extended to use haplotypic markers instead of marker data to fully use the available multi-allelic information at different genotyped loci.

It should be pointed out that our prediction relies on probabilistic models, which are in no way related to the mechanistic ODE-based model used for generating the data set. In essence, none of our models is therefore using the “true model”, which is the usual case when handling real data sets.

A potentially more challenging question lies in the number of genes in the network. As we have just pointed out, the analysis of a large number of genes requires large sample sizes, at higher costs. When dealing with GRN with thousands of genes and only a few hundreds of individuals in the population, the ultra-high dimension limit linking the sample size, the number of genes and the network sparsity is hit so that even sparse models can not be faithfully recovered anymore [41]. In the three formerly cited papers ([37]–[39]), if the number of parents/regressors associated to each gene was to exceed 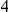, the estimation would theoretically be impossible. Bootstrap techniques might help in providing sparse robust estimates in such settings [42]–[44]. A prior selection of relevant genes, using genes that are differentially expressed or selecting genes known to play a role in the biological process under study, could considerably improve GRN inference. The risk here is that if an important variable (*e.g.* integrative hub) is missing in the data set, confounding effects will likely lead to false positive edge predictions even when combining several methods into a powerful meta-analysis. The use of hidden variables, that could account for unmeasured gene expressions, has shown limited performances when the number of genes in the network is high (over a few tens of genes) ; interesting preliminary results can be found in [45], [46]. There is a substantial need for methodological developments in this direction.

Following the added value of integrating several inference methods, a natural way to improve predictions would be to include additional inference methods which would complement the methods we used in the present study. Causality inference is probably the area where our current combination of inference tools could benefit from additional contributors. Indeed, the linear regression inferences essentially ignore causality, while Bayesian networks are able to predict causality when no Markov equivalence ambiguity appears. One should ideally be able to actively exploit the fact that the seed for causality from polymorphism to expression is known *a priori*. Before this, different existing inference techniques, such as kernel methods [47] and Random Forests [48], which have already been used in similar contexts [49], [50], would be excellent candidates.

## Materials and Methods

### Notations and data simulation

The data sets provided by the DREAM5 Challenge 3 organisers are available at http://wiki.c2b2.columbia.edu/dream/index.php/D5c3.

Directed networks of 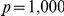 genes were generated according to a “modular scale-free topology” [18]. After the challenge, the organisers gave additional information on the generation process: networks were simulated with a power law (scale free) out-degree distribution, but an exponential in-degree distribution. Moreover, simulated networks were modular. Fifteen such networks were generated and the distribution parameters were chosen so that the total number of edges range from 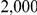 (Network ‘1’ category) to 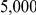 (Network ‘5’ category). Each network was associated to a specific population size 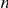 of either 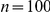 (sub-challenge A100), 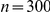 (sub-challenge A300) and 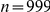 (sub-challenge A999). In all cases, the sample size 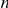 was smaller than the total number of genes 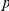 and the 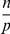 ratio, which is important for estimation, varied from 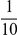 for sub-challenge A100 to 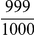 for sub-challenge A999.

For each network and each population size, genotypes for 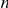 RILs with 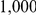 bi-allelic markers evenly distributed on 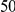 chromosomes were simulated using linkage information. Each RIL was an homozygous mosaic of paternal and maternal alleles. Parental alleles were different all along the genome. Each marker polymorphism was assumed to be associated with a single gene mutation located either in the promoter region (probability 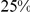) or in the coding region (probability 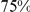). A polymorphism in the promoter region of a gene affects its basal transcription rate, leading to a ‘*cis*-like’ regulatory effect on the gene activity, while a polymorphism in the coding region affects the strength of the effect of the gene on its targets in the network, leading to a ‘*trans*-like’ effect. The marker data for RIL 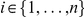 and gene 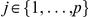 is denoted 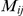 and has value 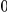 or 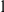. The genotype matrix 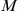 is hence a 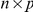 matrix with 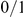 entries.

Gene expression levels were simulated at steady state of a dynamical system represented by a set of ODEs (see exact formula in [18] and details in [51]). These ODEs account, *via* different parametrisations, for different intensities in activation or repression effects, genetic variant influences and additional noise. The expression data matrix 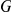 consists in a 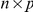 matrix.

**Figure 8:**
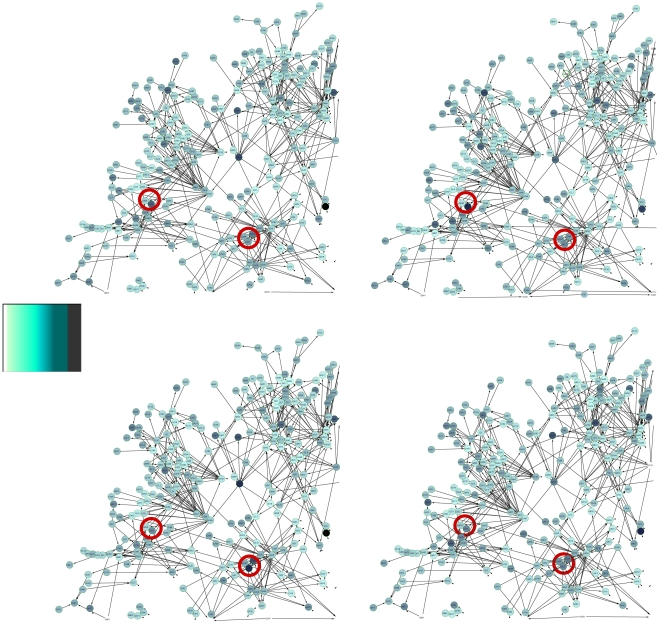
Graphical representation of expression data on a subpart of Network1-A999 for four individuals. Node colour represents simulated gene expression level (in green scale, light for small values and dark for high values) for individuals 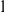 (upper left), 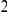 (upper right), 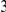 (bottom left) and 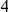 (bottom right). Red circles highlight two spots in the network that vary due to different underlying marker polymorphisms.

Polymorphisms between RIL individuals define multifactorial perturbations. Each allelic combination defines a different parametrisation in the ODE model, with the same network skeleton. In addition to random term effects that represent technical or biological variability, this provokes changes in the observed gene expression patterns from one individual to another that in turn influence each other according to the causal network. The observed values 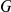 were those obtained at steady-state of the complex system. Figure 8 depicts such observed patterns for four individuals (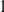 to 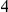) of the population for Network1-A999 of the challenge.

The goal of the challenge was to reconstruct the network that gave birth to the observed measures as a list of edges sorted according to a relative “confidence” score. This score was only used for ranking edges.

We now present our strategy to reconstruct the networks namely the preprocessing of the data, the probabilistic graphical models that we implemented, their post-processing and the meta-analysis that was carried out to make the best out of the different modelling approaches.

### Bayesian networks

Our first statistical modelling of the data relies on a directed graphical model known as Bayesian networks. Our model captures expression levels and genetic data in discrete variables, related through conditional probability tables capturing regulating and polymorphism effects, including possibly non-linear effects. The structure and parameters of the underlying graph were estimated using a score-based structure learning algorithm similarly to what was done in [13] in the context of pure expression data analysis. The precise score, discretisation policy, and algorithm used are described below.

#### Bayesian networks and structure learning with the Dirichlet score

A Bayesian network denoted by 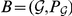 is defined by a directed acyclic graph 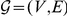 with nodes representing 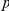 random discrete variables 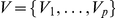, linked by a set of directed edges 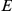, and a set of conditional probability distributions 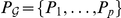. The variables involved in each conditional probability table 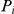 are defined by the directed acyclic graph: 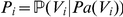, where 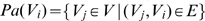 is the set of parent nodes of 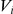 in 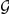. A Bayesian network 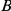 represents a joint probability distribution on 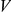 such that:
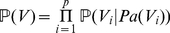
(1)


Learning the structure of a Bayesian network consists in finding a directed acyclic graph 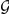 maximising 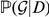 where 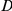 represents the observed data. We have:
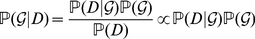
(2)


The first term 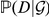 of Equation 2, is called the marginal likelihood. The Bayesian Dirichlet score (BDeu, where ‘eu’ stands for Equivalent Uniform) gives the same score for Markov equivalent Bayesian networks and assumes a uniform prior on the conditional probability parameters. It is defined by the following expression: 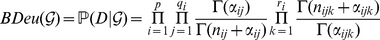
with 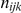, the number of occurrences of the configuration 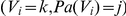 in the 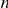 samples, 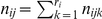, 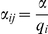, and 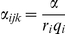, where 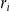 is the domain size of variable 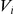 and 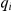 is the size of the Cartesian product of the 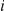 parent domains (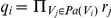). The BDeu score requires a specific value for 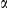, called the equivalent sample size, which, in practice, is often arbitrarily set to one. However, [52], [53] established the sensitivity of the BDeu score with respect to this parameter: the connectivity of the inferred DAG increases with a growing 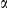. [53] suggested a way to compute an optimal 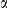 value, making the assumption that 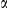 is smaller than the sample size. Following this idea, we defined a range of 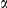 values starting from its maximum value, set to the largest sample size (*i.e.* 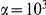), and decreasing it on a logarithmic scale. In our experiments, we varied 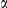 in the range 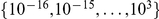 in order to get 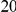 networks, from a very sparse to a denser structure. This defined the 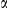-grid for the Bayesian network approach. We defined the score of an edge using majority voting on these graphs (see below). Without any additional information, a uniform probability over all possible DAGs was assumed in Equation 2.

#### Bayesian network modelling and discretisation policy

The set of discrete random variables 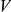 was composed of one variable per gene-activity, denoted 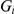, and one variable for each genetic marker, denoted 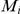, for all 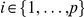 with 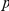 the number of genes (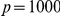). Following challenge 3A assumption, each gene, with expression 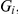 was associated with a single genetic marker 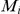. Since we used discrete BN, we had to discretise 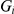. As shown in [54], for the same score-based structure learning algorithm, the choice of a discretisation method can dramatically modify the quality of the inferred network. Instead of choosing a single discretisation method, we chose an adaptive method depending on the type of gene-activity distribution for each gene. Observing complex distributions in the data sets, we distinguished two types of distributions. If we detected a unimodal (normal-like) distribution, we used an adapted 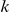-means algorithm to obtain a three-class discretisation, which also ensured a minimum class size (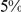 sample size) and a maximum size for extreme classes (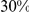). In the case of a multimodal distribution, we used the more general framework of Gaussian mixture models to find a maximum of four classes. Since the BDeu score depends on domain sizes, we tuned the parameters of our discretisation method to favour a four-class discretisation so that most of the 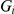 variables had the same domain size.

#### Structure learning and restricted search space

Learning Bayesian network is an NP-hard problem with a super-exponential search space of potential DAG structures [33] and even a greedy search heuristic method can be very time consuming when the number of variables 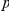 is large. In order to get reasonable computation times and also take into account biological knowledge, we reduced the search space by several assumptions.

A preliminary analysis of variance was used to predict *cis*-regulatory markers: detected positive markers (Bonferroni corrected 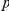-value 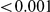) were those giving the most significant signal in a 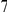 marker-width window, centred on the gene, to avoid false marker influence due to genetic linkage.

We used this *cis*-effect information to constrain structure search: since each *cis*-marker 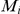 had an effect on its associated gene activity 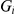 only, we constrained our model to use an 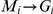 edge and forbade other edges outgoing from 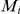. In the opposite case, when the marker 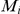 was not detected as *cis*-regulatory marker we only forbade the 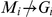 edge.

Following the approach of [55], for each gene expression 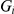, we selected a list of candidate parents composed of genes 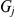 (resp. markers 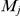) with a contribution to BDeu 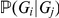 (resp. 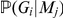) assuming a single parent 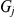 (resp. 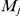) greater than 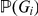 assuming no parents. Moreover, due to the fact that markers in the same chromosome region had a tendency to be selected together because of linkage correlations, we chose the best marker in a 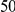 cMorgan sliding window. We did not try to learn edges between marker variables since it is useless for our purpose.

We used the structure learning algorithm ‘greedy hill-climbing’ of Banjo [56]. We started from an empty DAG and fixed a maximum number of parents to 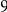 to avoid overwhelming computational costs, in order to find the best DAG locally maximising Equation 2 for each value in the 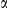-grid.

The directed edges from the resulting 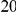 DAGs learnt for the 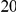 different values of 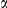 were directly mapped onto genes to define a network relating the 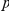 genes: an edge from 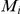 to 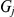 in the learnt structure created an edge from 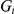 to 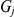 in the network. So, despite the fact that the underlying graphical model can only represent an acyclic directed structure, the final network may contain cycles. We computed the frequency of every directed edge in the inferred gene networks obtained by different values of the equivalent sample size 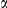. This allowed us to perform a simple majority vote; directed edges were sorted based on their frequency, breaking ties by using average influence scores as defined in [57].

### Structural equation modelling

This section first presents the structural equation model used to describe relationships among variables and the penalisation techniques that allowed for simultaneous parameter estimation and variable selection. We then explain how we implemented them in practice.

In the framework of Structural Equation Models (SEM), one response (or dependent) variable 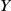 is assumed to depend upon 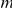 regressors 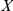
's with linear dependency in the parameters:



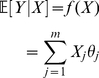
(3)


Equation 3 is linear in parameters 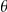 that are unknown and need to be estimated. Explanatory variables 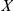 can be quantitative or qualitative.

Having observed 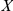 and 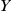 for a sample of size 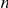, the usual estimation procedure is the ordinary least square (OLS) method which minimises the residual sum of squares (RSS):



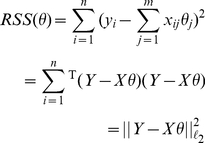
(4)where 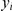 and 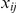 are the observed values of 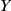 and 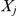 for the 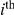 individual.

Differentiating Equation (4) with respect to 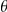 leads to the unique least square estimate



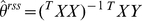
(5)


On data sets where 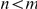, as in our case, 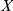 may be not full rank. 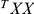 is then singular and the estimate in Equation (5) can be replaced by: 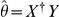 where 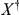 is the Moore-Penrose pseudo inverse of 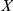. Beyond intensive computation, another difficulty arises when eigenvalues of 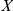 close to 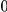 cannot be estimated precisely enough because of numerical instability, inducing large uncertainty about 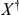 and consequently 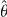. Ultimately, the unbiased estimate 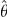 is not worth the effort because of the induced variance in coefficient estimates or predictions.

Since our goal was to obtain an interpretable (*i.e.* reasonable number of significant explanatory variables) and stable (*i.e.* small changes in data should have low impact on analysis results) model that leads to as accurate as possible predictions, we allowed some bias-variance trade-off using regularization. One approach to regularization is to introduce a constraint on regression coefficients. For example ridge regression minimises the RSS in Equation 4 imposing that 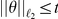 for some 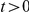. The smaller 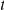, the greater the level of shrinkage of regression coefficients. Conversely, larger values of 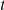 allow more complex models that are penalised if they do not bring enough gain into the RSS. The model selection problems therefore come down to choosing appropriate values for 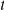. However, ridge regression does not select variables: every regression coefficient is shrunk but not set to 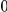 so the model is not really simpler. Direct variable selection procedures, such as “best subset” tackle the issue of a huge number of regressors included in the model but as a discrete process they can be subject to a high variance in the produced estimates.

Among the many possible techniques to achieve variable selection in a stable model, we chose to focus on the Lasso [25] and on the Dantzig Selector [26]. Other penalization techniques (for example see [43], [58], [59]) are known to be more suitable for high dimensional data that have inherent inner collinearity, but need additional parameter tuning. Our goal was first to focus on simple, efficient but powerful techniques in order to assess their merits on this problem.

#### Lasso penalised regression

The Lasso is very similar to ridge regression. It also minimises the RSS but allows for deviation up to a penalty term controlled by a constraint on the 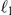 norm of parameters 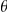 (instead of the 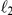-norm for ridge regression). The Lasso automatically selects variables and continuously shrinks their associated regression coefficients. Depending on the penalization strength, it enforces an increasing number of parameters to be 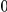. Lasso estimates are defined as follows:



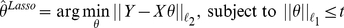
(6)or equivalently (Lagrangian transform):



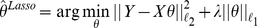
(7)


While Equation (6) explicits the constraint on the parameters norm, Equation (7) introduces the penalty parameter 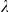: the larger 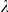, the greater the amount of shrinkage, and the simpler the selected model will be. More precisely, 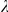 is an upper bound on the correlation between regressors not included in the model and the regression residual. Interpreting 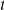 of Equation (6) is also possible by considering 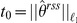. Hence setting 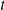 to 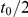 roughly shrinks active coefficients in the regression by 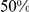 [44]. The Lasso solutions do not vary equally upon input scales. Standardisation of the inputs settles this problem. For the Lasso (and for the Dantzig selector below), we therefore standardised the input regressors.

Solving Equation (7) is a quadratic programming problem but efficient algorithms exist for computing the entire solution path as 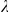 varies. We used the the Least Angle Regression (LAR, [34]) algorithm available in the glmnet package version 1.4 [60] and implemented in R (version 2.11.0, http://www.r-project.org/).

In the challenge, confidence scores had to be assigned to inferred edges, so we did not use a model selection criterion but instead created a score reflecting the importance of the explanatory variable. This score was the frequency for this variable to be included in the model for different values of the penalization parameter. This could be done along the entire LAR solution path. We used a fixed grid of 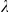 values. For comparability with the Dantzig selector and BN approaches, we used a grid of 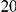 evenly spaced values for 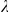, ranging from 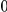 (no penalization) to 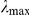, the smallest value of 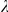 that prevents any regressor to be included in any regression.

#### Dantzig selector

The Dantzig selector [26] is a recent regression method which, as the Lasso approach, relies on the 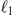 norm of the parameters to capture model complexity. In its standard description, the Dantzig selector minimises the 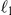 norm of the parameters subject to constraints bounding the absolute value of the correlation between residuals and explanatory variables. Similarly to the definition of the Lasso given in Equations (6) and (7), the Dantzig Selector is:



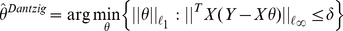
(8)where 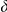 is a bound on the correlation between the residual vector and each explanatory variable. With no bound (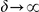), the Dantzig selector estimates all coefficients to zero, because of the minimised 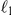 norm. With the strongest bound (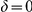), Dantzig enforces a zero correlation between residuals and explanatory variables, a condition which is also satisfied by ordinary least square estimates (as it is equivalent to enforcing a zero derivative of the squared error term minimised in OLS regression). Equation (8) can be written in its dual form:



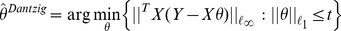
(9)


This writing is similar to the Lasso of Equation (6), replacing the RSS by the maximum varying component of its gradient.

As initially shown in [26], the Dantzig selector is able to produce an accurate estimate in the 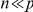 context with a bounded error term, provided that the model is actually sparse. The Dantzig selector also has the property that it reduces regression to linear programming, a polynomial optimisation problem [61] for which efficient dedicated solvers exist. Recently, [35] showed that the Lasso and the Dantzig selector share similar properties: the Lasso estimate automatically satisfies Dantzig correlation constraints, and similar error boundaries can be obtained in both cases (although with larger constant terms for the Lasso). For this reason, the Dantzig selector tends to be considered as extremely similar to the Lasso. However [44] noticed that coefficient regularization paths are quite smooth along Lasso solution while they can become irregular with the Dantzig selector.

To solve each regression problem, we generated a linear program (LP) as described in [26]. The generated LP was far from optimised. Beyond the 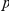 variables for vector 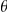, it included 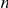 variables for the residual vector and 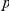 variables to encode boundaries on correlations. 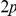 boundaries were used to effectively limit correlations and an extra set of 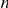 linear equalities encoding residual definition. The size of this encoding depended on 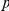 and 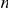 but could easily depend just on 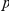 (by symbolic precomputation of the scalar product between residuals and explanatory variables).

By setting all parameters 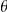 to 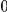 while minimising 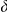, it is simple to compute the minimum value of 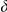 such that all regression coefficients are set to 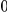 (denoted as 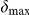). We then solved the Dantzig selector problem using the GPL linear programming solver glpk for 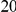 evenly spaced values of 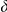 in 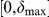 (for comparability reasons with the BN and Lasso approaches), providing a set of 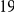 non-trivial estimates for the parameters in 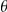.

#### Application of structural equation models to systems genetics data

We now show how we used penalised regressions to infer a GRN from the DREAM5 Challenge 3A data sets.

We regressed each gene expression level 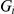 for 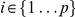 using as regressors every other gene expression level and every gene marker. This gene-by-gene approach ignores correlations and therefore corresponds to the minimisation of a specific penalised pseudo-likelihood [62]. Its main advantage is to reduce the whole penalised likelihood minimisation to 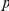 univariate penalised linear regressions.

Let 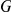 denotes the 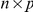 observed matrix of the gene expression levels and 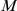 the 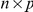 matrix of marker genotypes; the linear regression model for gene 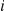 is:



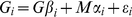
(10)

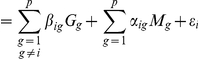
where 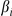 is the 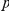-vector of linear effects of other expression levels on 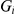 (forcing 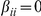 to avoid trivial self-regression), 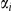 is the 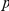-vector of linear effects of markers on 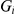 and 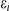 is the Gaussian residual error term.

To make the link with previous notations in this section, 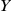 now iteratively becomes one of the 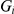 variables, 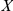 becomes the 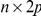 matrix 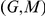 and the regression coefficients 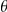 now become 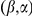. The network is then encoded in non-zero entries of estimated matrices 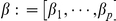 and 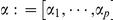. The only consistency condition is that 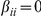 for all 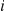.

From the estimated 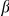 and 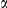 matrices, the gene-to-gene network was inferred. More precisely, when 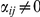 for some 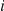 and 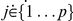, we inferred edge 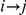 in the gene network and assigned it a count of 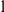. If both 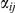 and 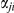 are equal to zero, then the 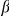 matrix was explored. If 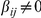 or 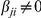, we inferred both edges 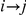 and 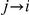 and assigned them a count of 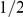. Finally we computed for each edge, the count mean in the chosen 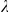-grid for Lasso or on the 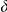-grid for Dantzig. This means that we put a high confidence level in directed edges inferred from marker to a gene expression level and that we inferred edges between gene expression data by symmetrising and halving their strength. This choice is somewhat arbitrary and can certainly be improved, as we commented in the discussion.

### Meta-analysis: integrating several network inference methods

We used a Fisher's Inverse Chi-Square meta-test [63] to combine the BN, Lasso and Dantzig predicted networks. This meta-test was initially introduced to combine the test values obtained from independent experiments. It consists of summing the opposite of the logarithm of the corresponding 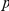-values.

In the output data for the DREAM challenge, we considered the “reliability” parameter as 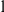 minus 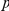-value since it is a measure of uncertainty in the 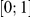 range. We denoted 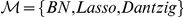 and 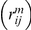 the edge reliability parameters associated to the method 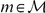. We then computed the sum 
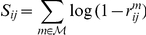



The meta-analysis picks up edges from the different approaches and computes a consensus ranking scheme that depends on individual scores of the methods and agreement between them.

Finally the meta-analysis edge reliability parameters are defined as 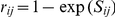 and were used to produce a ranked list a edges for each inferred network. Since the organisers limited the edge list length that could be submitted to 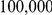 among the 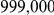 possible edges in each network (no self loops were considered), we arbitrarily cut the list according to the ranking when necessary. In practice, we never predicted more than 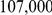 edges.

### Accuracy assessment: scoring methods

Once submitted to the DREAM5 challenge organisers, edge lists were compared both to (i) Gold Standard files, namely the correct list of edges used in simulated models and to (ii) the pool of all edges that were submitted by other participating teams.

The Gold Standard comparison allows to assess prediction accuracy based on two measures, namely the “area under the curve” (AUC) score for the Receiver Operating Curve (ROC *i.e.* true positive versus false positive rates) and the precision versus recall (PR) curve. The second comparison evaluates predictions on the basis of their intrinsic merit and on their ability to bring in specific predictions compared with the pool of all predicted edges.

Let TP, FP, FN and TN denote respectively the true positives (correctly inferred edges), false positives (edges inferred by mistake), false negatives (missed edges) and true negatives (correctly non-predicted edges), then (i) False positive Rate (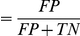, (ii) Precision 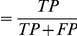 and (iii) Recall 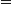 True positive rate 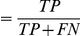. Notice that the orientation of edges is significant in the comparison so that an edge 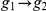 is not considered as correct if the true edge is 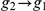.

The second comparison assesses the prediction of every method against the pool of all the predictions submitted by competing teams. It was used to produce 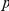-values that reflect how well each method performed in comparison to others. More precisely, the lower the 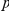-value for a team prediction AUC, the higher the probability that it could not be reached by a random network built by picking up edges (at the same rank) from the pool of all submitted networks. The 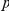-values for all 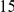 different networks were then 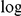-transformed and summed in absolute value. The higher the resulting score, the better the method performed over the challenge. A detailed description of the scoring scheme for the DREAM5 challenges can be found in [27].
